# Preoperative meloxicam versus postoperative meloxicam for pain control, patients’ satisfaction and function recovery in hip osteoarthritis patients who receive total hip arthroplasty: a randomized, controlled study

**DOI:** 10.1007/s10787-020-00718-2

**Published:** 2020-06-06

**Authors:** Lingyun Ren, Li Meng, Hong Yan, Wei Sun, Dan Yao

**Affiliations:** Department of Anesthesiology, The Central Hospital of Wuhan, Tongji Medical College, Huazhong University of Science and Technology, No. 26, Shengli Street, Wuhan, 430014 People’s Republic of China

**Keywords:** Hip osteoarthritis, Total hip arthroplasty, Preoperative, Meloxicam, Pain control

## Abstract

This study aimed to compare the analgesic effect, patients’ satisfaction, tolerance and hip-joint function recovery by preoperative meloxicam versus postoperative meloxicam in treating hip osteoarthritis (OA) patients receiving total hip arthroplasty (THA). 132 hip OA patients who underwent THA surgery were allocated into postoperative analgesia (POST) and preoperative analgesia (PRE) groups at a 1:1 ratio. In the PRE group, patients took meloxicam 15 mg at 24 h pre-operation, 7.5 mg at 4 h, 24 h, 48 h and 72 h post-operation; in the POST group, patients received meloxicam 15 mg at 4 h post-operation, then 7.5 mg at 24 h, 48 h and 72 h post-operation. Furthermore, postoperative pain, consumption of patient-controlled analgesia (PCA), overall satisfaction and adverse events were evaluated within 96 h post-operation; meanwhile, Harris hip score was assessed within 6 months post-operation. Pain VAS at rest at 6 h, 12 h, 24 h, and pain VAS at passive movement at 6 h, 12 h were decreased in PRE group compared to POST group. In addition, additional consumption of PCA and the total consumption of PCA were both reduced in PRE group compared to POST group. Additionally, overall satisfaction in PRE group was higher at 24 h, 48 h and 72 h compared to POST group. While Harris hip score was of no difference between POST group and PRE group at M3 or M6. Besides, no difference in adverse events incidence was found between the two groups. In conclusion, preoperative meloxicam achieves better efficacy and similar tolerance compared to postoperative meloxicam in hip OA patients post THA.

## Introduction

Osteoarthritis (OA) is a common degenerative joint disease and is one of the most predominant causes of pain and physical inactivity in the worldwide population (Aresti et al. 2016; Skou et al. 2019). Among the total OA cases, articulatio coxae, also named as hip joint, is the second most frequently involved joint, resulting in chronic pain in patients’ outer hip, groin, and sometimes extends to the knee, and the pain often aggravates as the disease progresses (Aresti et al. 2016). Therapeutic modalities for hip OA patients include physical exercise, weight management, non-steroidal anti-inflammatory drugs (NSAIDs) or other analgesics, sodium hyaluronate injection and surgery (Migliore and Anichini 2017; Murphy et al. 2016; Skou and Roos 2019). Among all the treatment options mentioned above, total hip arthroplasty (THA) surgery is the gold standard for hip OA patients with severe disease conditions with satisfactory efficacy in improving the function of hip joint (Shon et al. 2019).

Reducing pain after THA surgery and maintaining the function recovery have always been the cornerstones of hip OA management in the clinical setting, as most of the patients would experience pain after receiving THA, and normally the pain could be moderate or even severe that notably harms the patients’ quality of life (Gan et al. 2003; Myles et al. 2000). Intravenous patient-controlled analgesia (PCA) is commonly applied for postoperative pain control in hip OA patients who receive THA, and several analgesics are frequently applied in PCA, such as the opioid drugs (Fan et al. 2018). However, excessive use of PCA may contribute to opioid drug overuse, which subsequently results in several adverse events, for instance, nausea and vomiting. Thereby, in hip OA patients who undergo THA, it is of note to explore a treatment strategy to achieve sufficient pain control and reduced adverse events at the same time. Meloxicam, a kind of NSAIDs, contributes a lot to the pain control in many diseases related to arthritis with good efficacy and tolerance (Park et al. 2014; Ruperto et al. 2005). In recent years, there have been accumulating studies revealing favorable efficiency of preoperative meloxicam in relieving pain post orthopedic surgeries (Hou et al. 2019; Shantiaee et al. 2017). Nevertheless, the efficacy of preoperative meloxicam for relieving pain in hip OA patients receiving THA is still unclear.

Thus, this randomized, controlled study aimed to compare the efficacy and safety of preoperative meloxicam versus postoperative meloxicam in treating hip OA patients who received THA.

## Materials and methods

### Patients

A total of 132 hip OA who scheduled to receive THA surgery, between Jan. 2017 and Dec. 2018 were consecutively enrolled in this randomized, controlled study. The inclusion criteria were: (1) diagnosed as hip OA by clinical and imaging findings; (2) age above 18 years; (3) appropriate and scheduled to receive THA surgery; (4) American Society of Anesthesiology (ASA) physical status I–II. Meanwhile, the exclusion criteria were: (1) hypersensitivity to NSAIDs; (2) usage of corticosteroid medication or chronic opioids within 3 months; (3) usage of analgesic drugs within 7 days; (4) uncontrolled hypertension during rest at two repeated measurements; (5) severe heart, kidney or liver dysfunction (6) recent major trauma or systemic infection within 3 months; (7) history of hip surgery; (8) history of bleeding or coagulation disorders; (9) history of gastrointestinal ulceration or dyspepsia.

### Ethics approval

This study was approved by the Ethics Committee of our hospital, and was conducted in line with the Declaration of Helsinki. Meanwhile, all patients provided written informed consents before enrollment.

### Sample size calculation

Based on the primary outcome of pain visual analogue scale score (VAS) at rest, we assumed that pain VAS at rest was 2.0 ± 0.5 and 2.3 ± 0.5 in the preoperative analgesia group (PRE group) and postoperative analgesia group (POST group), respectively. Subsequently, according to a power of 90% (*β*), a 5% level of significance (*α*) with double side and a sample size ratio of 1:1, the smallest sample size was required to be 60 in each group. Meanwhile, considering a 10% drop-out rate, the smallest sample size was set to 66 in each group with a total of 132 in the study.

### Randomization

After enrollment, 132 hip OA patients were randomly allocated to PRE group (*N* = 66) and POST group (*N* = 66) as a 1:1 ratio based on blocked randomization method with block length of 6. The randomized code was generated by SAS 9.0 software (Statistical Analysis System, USA), and the execution of randomization was conducted by a third company (H&J CRO International, Inc., Shanghai, China).

### Treatment

After randomization, in PRE group, patients received meloxicam (Boehringer Ingelheim, German) as follows (Shao et al. 2019): 15 mg (oral) at 24 h pre-operation, 7.5 mg (oral) at 4 h, 24 h, 48 h and 72 h post-operation, respectively; while in POST group, patients received meloxicam (Boehringer Ingelheim, German) as follows (Shao et al. 2019): 15 mg (oral) at 4 h post-operation, then 7.5 mg (oral) at 24 h, 48 h and 72 h post-operation, respectively. Besides, as a routine, all patients in both two groups received 0.1 mg fentanyl (Yichang Humanwell Pharmaceutical Co., LTD, China) and 6 mg tropisetron mesylate (Qilu Pharmaceutical Co., LTD, China) by intravenous injection as a loading dose of analgesia, followed by the application of intravenous PCA for 48 h post-operation. The PCA contained 100 mL solution complemented with 1 mg fentanyl (Yichang Humanwell Pharmaceutical Co., LTD, China), 50 mg tramadol (Qianjiang Pharmaceutical Co., LTD, China) and 6 mg tropisetron mesylate (Qilu Pharmaceutical Co., LTD, China), with a basal rate of 1.0 mL/h, a lock-out time of 15 min and a bolus dose of 0.5 mL.

### Assessments

Pain VAS at rest (0–10 point, 0 no pain, 10 worst pain) and pain VAS at passive movement (0–10 point, 0 no pain, 10 worst pain) were evaluated at the enrollment (Pre), 6 h (h), 12 h, 24 h, 48 h, 72 h, and 96 h post-operation. Meanwhile, the consumption of PCA was recorded during 96 h post-operation. Overall satisfaction (0–10 point, 0 worst satisfaction, 10 best satisfaction) was evaluated at 24 h, 48 h, 72 h, and 96 h post-operation. Besides, the adverse events (AEs) during 96 h intervention period were recorded. After the 96 h intervention period, patients were further followed up for 6 months (M), and Harris hip score was evaluated at the enrollment (Pre), M3 and M6.

### Statistics

Statistical analysis was performed using SPSS Software Version 22.0 (IBM, USA), figures were made using GraphPad Software Version 7.00 (GraphPad, USA). Data were mainly exhibited as mean ± standard deviation or count (%). Comparisons between two groups were detected by the *t* test or Chi-square test. *P* < 0.05 was considered as significant.

## Results

### Study flow

**Fig. 1:**
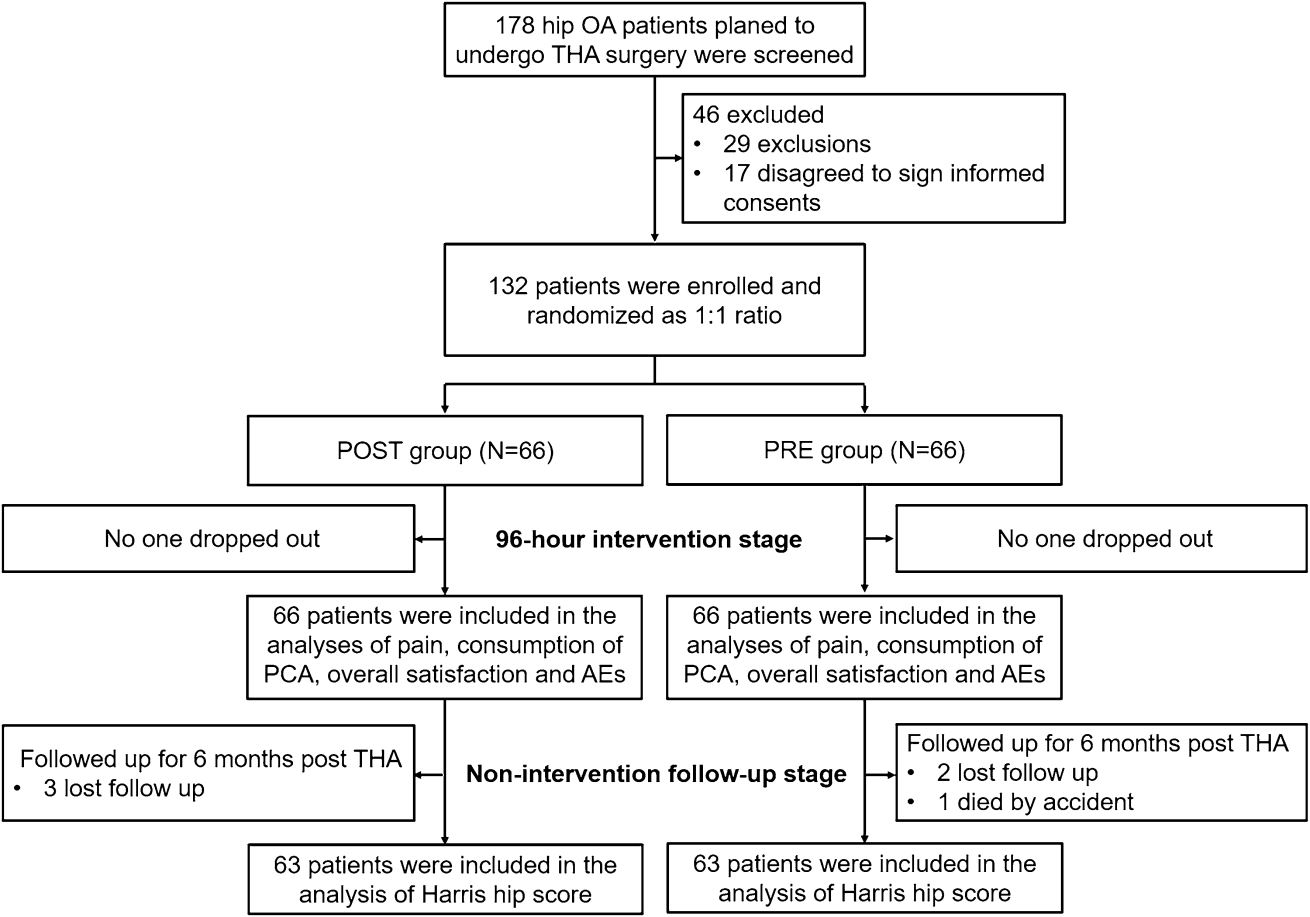
Study flow chart

A hundred and seventy-eight hip OA patients who planned to undergo THA surgery were screened, then 46 of them were excluded because of being incompatible with inclusions or meeting the exclusions (*n* = 29) and disagreement to sign the informed consents (*n* = 17) (Fig. 1). The remaining eligible 132 patients were enrolled and randomized as a 1:1 ratio into POST group (*n* = 66) and PRE group (*n* = 66), respectively. The study was separated into two stages: 96-h intervention stage and the non-intervention follow-up stage. In the 96-h intervention stage, no one dropped out from POST group or PRE group; all 66 patients in POST group and all 66 patients in PRE group were included in the analyses of pain, consumption of PCA, overall satisfaction and AEs. Subsequently, in the non-intervention follow-up stage, all patients were followed up for 6 months post THA; in POST group, there were three patients who lost follow-up; in PRE group, there were two patients who lost follow up and one patient who died by accident. Finally, there were 63 patients who were included in the analysis of Harris hip score in each group.

### The characteristics of patients

No difference in patients’ characteristics was found between POST group and PRE group (all *P* > 0.05) (Table 1). The patients in our study had a mean age of 64.7 ± 7.9 years in POST group and 63.1 ± 8.4 years in PRE group. There were 29 (44.0%) males and 37 (56.0%) females in POST group, and 24 (36.4%) males as well as 42 (63.6%) females in PRE group, respectively. In addition, the mean BMI was 23.6 ± 2.2 kg/m^2^ in POST group and was 23.1 ± 2.4 kg/m^2^ in PRE group. Besides, pain VAS at rest and pain VAS at passive movement were 4.9 ± 1.2 and 6.2 ± 1.4 in POST group, and were 4.8 ± 1.3 as well as 6.3 ± 1.3 in PRE group, respectively. Moreover, the Harris hip score in average was 6.2 ± 1.4 in POST group and was 6.3 ± 1.3 in PRE group.

**Table 1:** Patients’ characteristics

Parameters	POST group (*N* = 66)	PRE group (*N* = 66)	*P* value
Age (years), mean ± SD	64.7 ± 7.9	63.1 ± 8.4	0.310
Gender, No. (%)			0.375
Male	29 (44.0)	24 (36.4)	
Female	37 (56.0)	42 (63.6)	
BMI (kg/m^2^), mean ± SD	23.6 ± 2.2	23.1 ± 2.4	0.253
Pain VAS at rest, mean ± SD	4.9 ± 1.2	4.8 ± 1.3	0.647
Pain VAS at passive movement, mean ± SD	6.2 ± 1.4	6.3 ± 1.3	0.672
Harris hip score, mean ± SD	42.6 ± 11.5	41.3 ± 10.7	0.502

Comparison was determined by t test or Chi-square test

*POST group* postoperative analgesia group, *PRE group* preoperative analgesia group, *SD* standard deviation, *BMI* body mass index, *VAS* visual analogue scale

### Comparison of postoperative pain control

**Fig. 2:**
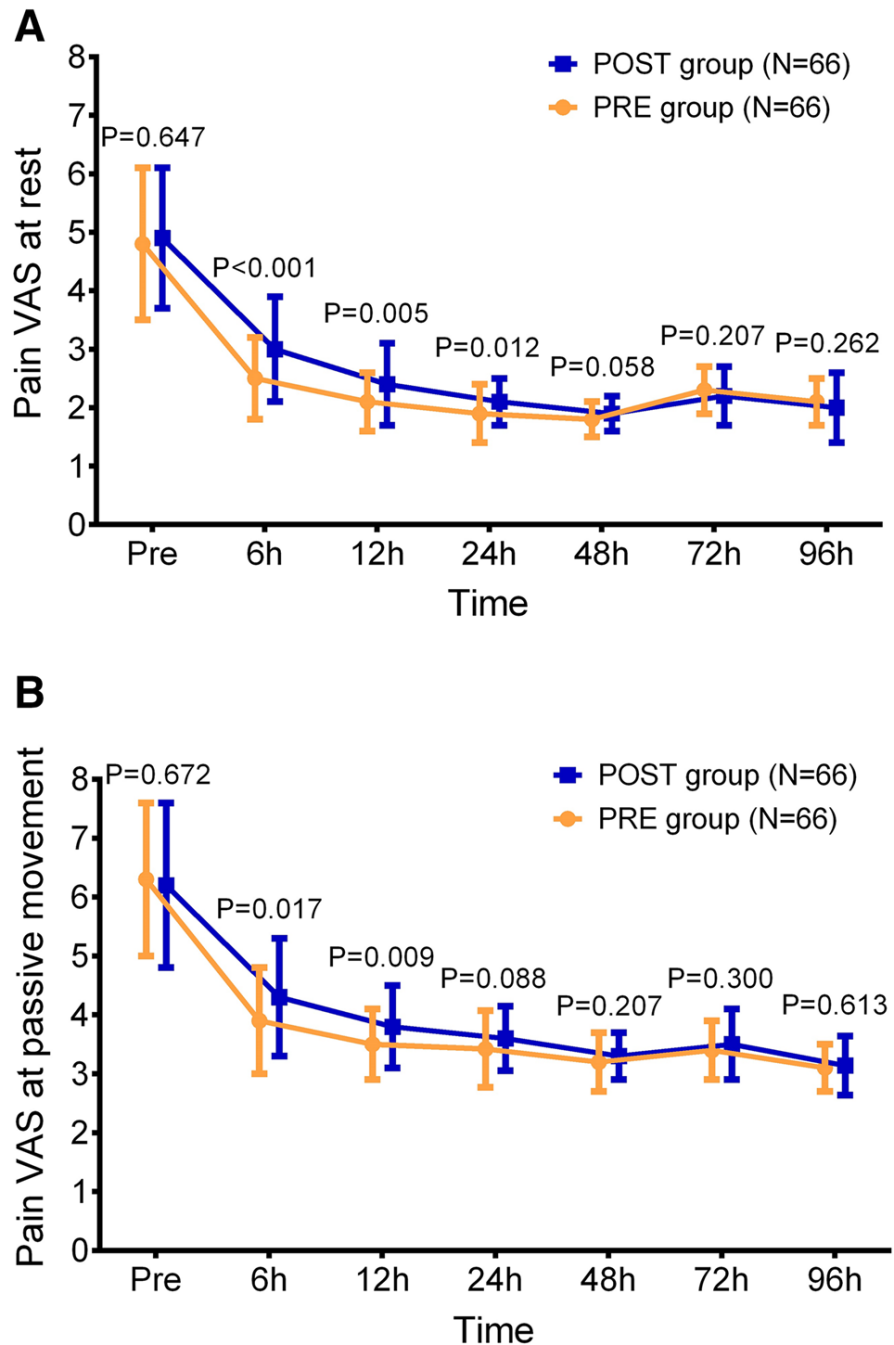
Pain VAS at rest and pain VAS at passive movement. The comparison of pain VAS at rest (**a**) and pain VAS at passive movement (**b**) at Pre, 6 h, 12 h, 24 h, 48 h, 72 h and 96 h between the POST group and the PRE group. *VAS* visual analogue scale score, *Pre* enrollment, *POST* postoperative analgesia group, *PRE* preoperative analgesia group

**Fig. 3:**
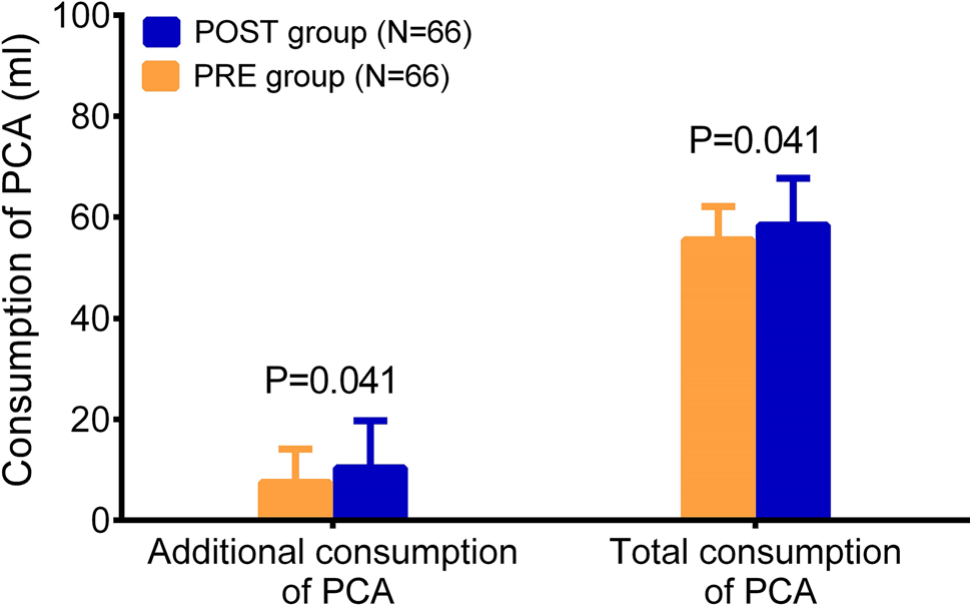
Consumption of PCA. The comparison of additional consumption of PCA and total consumption of PCA between the POST group and the PRE group. *PCA* patient-controlled analgesia, *POST* postoperative analgesia group, *PRE* preoperative analgesia group

In the 96-h intervention stage, pain VAS at rest and pain VAS at passive movement gradually decreased over time in both POST group and PRE group. The pain VAS at rest in PRE group was decreased at 6 h (*P* < 0.001), 12 h (*P* = 0.005) and 24 h (*P* = 0.012), while was of no difference at 48 h (*P* = 0.058), 72 h (*P* = 0.207) or 96 h (*P* = 0.262) compared to the POST group (Fig. 2a). As for pain VAS at passive movement, it was declined in PRE group at 6 h (*P* = 0.017) and 12 h (*P* = 0.009), but did not vary at 24 h (*P* = 0.088), 48 h (*P* = 0.207), 72 h (*P* = 0.300) or 96 h (*P* = 0.613) compared to POST group (Fig. 2b). Moreover, the additional consumption of PCA was decreased in PRE group than that in POST group (*P* = 0.041), and the total consumption of PCA was also reduced in PRE group compared to POST group (*P* = 0.041) (Fig. 3).

### Comparison of patients’ satisfaction

**Fig. 4:**
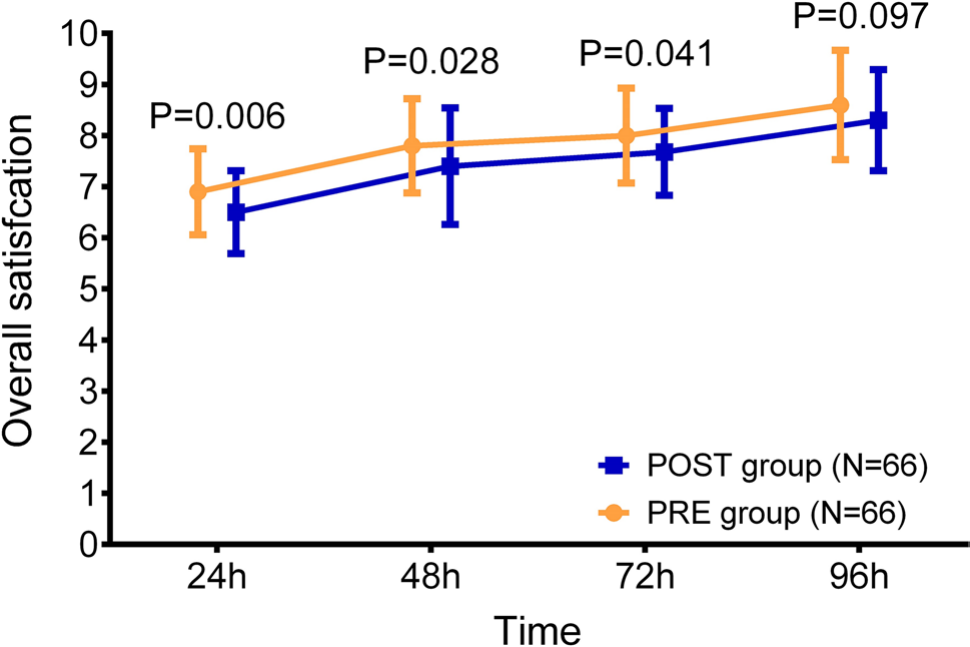
Patients’ overall satisfaction. The comparison of overall satisfaction at 24 h, 48 h, 72 h and 96 h between the POST group and the PRE group. *POST* postoperative analgesia group, *PRE* preoperative analgesia group

In the 96-h intervention stage, the patients’ satisfaction post-operation constantly increased along with time in both POST group and PRE group. More importantly, the overall satisfaction in the PRE group was elevated at 24 h (*P* = 0.006), 48 h (*P* = 0.028), and 72 h (*P* = 0.041) but was of no difference at 96 h (*P* = 0.097) compared to the POST group (Fig. 4).

### Comparison of adverse events

In the 96-h intervention stage, no difference in the adverse events incidences was found between the PRE group and the POST group, which included nausea (*P* = 0.333), vomiting (*P* = 0.572), constipation (*P* = 0.730), urinary retention (*P* = 0.310), drowsiness (*P* = 0.541), dizziness (*P* = 0.541) and others (*P* = 1.000) (Table 2).

**Table 2:** Adverse events

Parameters	POST group (*N* = 66)	PRE group (*N* = 66)	*P* value
Nausea, No. (%)	21 (31.8)	16 (26.6)	0.333
Vomiting, No. (%)	8 (12.1)	6 (9.1)	0.572
Constipation, No. (%)	4 (6.1)	5 (7.6)	0.730
Urinary retention, No. (%)	3 (4.5)	1 (1.5)	0.310
Drowsiness, No. (%)	2 (3.0)	1 (1.5)	0.541
Dizziness, No. (%)	1 (1.5)	2 (3.0)	0.541
Others, No. (%)	3 (4.5)	3 (4.5)	1.000

Comparison was determined by Chi-square test

*POST group* postoperative analgesia group, *PRE group* preoperative analgesia group

### Comparison of hip joint function recovery

**Fig. 5:**
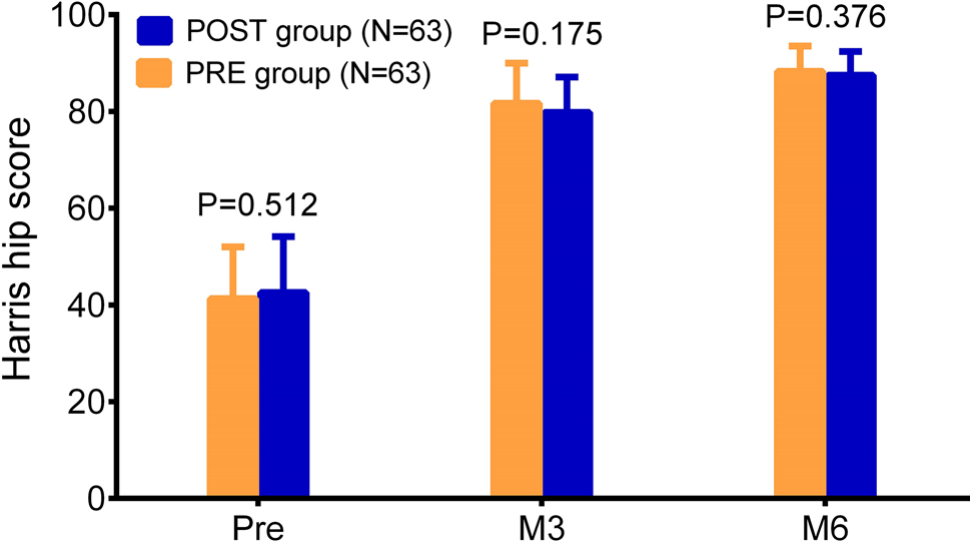
Harris hip score. The comparison of Harris hip score at Pre, M3 and M6 between the POST group and the PRE group. *Pre* enrollment, *M3* 3 months, *M6* 6 months, *POST* postoperative analgesia group, *PRE* preoperative analgesia group

In the non-intervention stage, the Harris hip score was elevated deliberately over time within 6 months in both POST group and PRE group. Then the results displayed that the score at M3 (*P* = 0.175) and M6 (*P* = 0.376) was similar between PRE group and POST group (Fig. 5).

## Discussion

In the present study, the effect of preoperative meloxicam versus postoperative meloxicam regarding postoperative pain control, patients’ satisfaction, adverse events and recovery of hip joint in hip OA patients who received THA were assessed. Then the findings illustrated that in hip OA patients who received THA, compared to postoperative meloxicam: (a) preoperative meloxicam was superior at reducing pain VAS at rest and pain VAS at passive movement; in addition, it also decreased additional and total consumption of PCA; (b) preoperative meloxicam elevated overall satisfaction; (c) preoperative meloxicam did not increase the incidence of adverse events; (d) preoperative meloxicam had no effect on improving the Harris hip score.

Meloxicam has been used for postoperative pain control for a long time, mostly in patients with arthritis, moreover, the preoperative use of meloxicam has been elucidated by several studies as a non-inferior modality compared to postoperative meloxicam for controlling the pain in patients with osteoarthritis post-surgery. A recent phase III randomized, multicenter, double-blind, placebo-controlled trial elucidates that meloxicam decreases the opioid use and does not increase the adverse events compared to placebo in patients with moderate-to-severe pain after major orthopedic surgeries (Sharpe et al. 2020). Another phase III, randomized, placebo-controlled study also reveals that meloxicam reduces opioid use and is evenly tolerable compared to placebo in patients with mild to severe pain after major orthopedic surgeries (Bergese et al. 2019). These studies uncover a good efficacy of meloxicam in controlling postoperative pain and its satisfactory tolerance in patients after various orthopedic surgeries. More importantly, in regard with the preoperative use of meloxicam, a previous study illuminates that preoperative meloxicam reduces pain VAS at rest, pain VAS at flexion, Physician's Global Assessment (PGA) score in knee OA patients who receive total knee arthroplasty (TKA) compared to postoperative use of meloxicam (Shao et al. 2019). In addition, another study reveals that early preoperative use of meloxicam diminishes pain VAS, PGA score, consumption of pethidine use compared with postoperative use of meloxicam in knee OA patients after arthroscopic knee surgery (AKS) (Hou et al. 2019). Furthermore, a prior study illustrates that very early preemptive meloxicam is more effective regarding reducing pain VAS at rest, pain VAS at flexion, and improving PGA score compared to early preemptive meloxicam as well as postoperative meloxicam in knee OA patients who receive AKS (Yuan et al. 2019). These findings all indicate a favorable efficiency and non-inferior tolerance of preoperative meloxicam in relieving postoperative pain in OA patients who receive orthopedic surgeries.

As for hip OA patients undergoing THA, the existed studies are very limited, and they all focus on researching the effect of preoperative meloxicam on preventing blood loss and heterotopic ossification in patients with hip OA after THA (Legenstein et al. 2003; van der Heide et al. 2004; Weber et al. 2003). To the best knowledge of ours, no study has been done to assess the effect of preoperative meloxicam on postoperative pain relieving in hip OA patients who receive THA. In the present study, we found that preoperative meloxicam reduced pain VAS at rest, pain VAS at passive movement and consumption of PCA compared to postoperative meloxicam in hip OA patients receiving THA. The possible explanations might include: patients in PRE group were treated with preoperative oral meloxicam and postoperative oral meloxicam, while patients in POST group were treated with only postoperative oral meloxicam. Therefore, in patients treated with preoperative meloxicam, the drug probably reached the concentration of stable state more quickly than patients treated with postoperative meloxicam; consequently, meloxicam could diminish painful stimulus and prevent the transmission of nerve impulse to the central nervous system more rapidly, which to some extend enabled a more favorable analgesic effect (Gates et al. 2005; Goncalves de Freitas et al. 2016). Besides, we also observed that the overall satisfaction was superior in patients receiving preoperative meloxicam compared to patients treated with postoperative meloxicam. And this result might derive from that the effect of pain control was more favorable in patients treated with preoperative meloxicam, which subsequently resulted in better patients’ satisfaction.

Meloxicam has always been a tolerable NASIDs for pain and postoperative pain control in OA patients. For example, a previous randomized controlled study reveals that meloxicam achieves similar adverse events incidence compared to placebo in patients with moderate to severe pain after major orthopedic surgeries, which consist of injection-site reactions, bleeding, cardiovascular, hepatic, renal, thrombotic, and wound-healing events (Bergese et al. 2019). As for the pre-operation use of meloxicam in OA patients after surgery, a study reports that early preoperative meloxicam injection achieves evenly adverse events incidence compared to postoperative meloxicam injection in knee OA patients post-AKS, including nausea, constipation, vomiting, dizziness and drowsiness (Hou et al. 2019). Another study also illuminates similar results, which discloses that no difference is found regarding adverse events proportions between knee OA patients receiving TKA treated with preoperative meloxicam administration and patients treated with postoperative meloxicam administration (Shao et al. 2019). In this study, we found that the adverse events were mostly nausea, vomiting, constipation, urinary retention, drowsiness, dizziness, and the incidence of these adverse events post-THA were similar between patients treated with preoperative meloxicam and patients treated with postoperative meloxicam. These results indicated that preoperative meloxicam was as tolerable as postoperative meloxicam in hip OA patients who received THA.

With reference to the improvement of hip joint function recovery, there is still no study reporting the effect of preoperative meloxicam on it in hip OA patients after THA. In this study, we discovered that the hip joint function assessed by Harris hip score was of no difference between hip OA patients receiving THA treated with preoperative meloxicam and hip OA patients receiving THA treated with postoperative meloxicam. This result might derive from that there were other factors affecting the recovery of hip joint function, for instance, the physical exercise. Moreover, this insignificance might also result from the relatively small sample size and short observational period in our study.

In this study, the limitations might include: (a) the sample size of 132 patients was relatively small, which possibly contributed to a less strong statistical power in the analyses; (b) in the 96-h intervention stage, the patients, clinicians or researchers were not blinded, thus, there might be bias in our study; (c) the follow-up period was 6 months in the non-interventional follow-up stage, which was a little short for assessing the long-term recovery of hip joint function.

Collectively, preoperative meloxicam is superior regarding postoperative pain control, patients’ satisfaction, and non-inferior in terms of safety as well as hip joint function recovery compared to postoperative meloxicam in treating hip OA patients who underwent THA.
